# Effect of *Eucommia ulmoides* Leaf Extract on Growth Performance, Carcass Traits, Parameters of Oxidative Stress, and Lipid Metabolism in Broiler Chickens

**DOI:** 10.3389/fvets.2022.945981

**Published:** 2022-07-28

**Authors:** Jiahao Yan, Ruizhi Hu, Baizhen Li, Jijun Tan, Ying Wang, Zhiyi Tang, Ming Liu, Chenxing Fu, Jianhua He, Xiaosong Wu

**Affiliations:** ^1^Hunan Collaborative Innovation Center for Utilization of Botanical Functional Ingredients, College of Animal Science and Technology, Hunan Agricultural University, Changsha, China; ^2^Animal Science and Technology College, Beijing University of Agriculture, Beijing, China

**Keywords:** broiler, *Eucommia ulmoides*, oxidative stress, meat quality, growth performance

## Abstract

*Eucommia ulmoides* bark has been traditionally used as a Chinese medicine to attenuate stress, but the leaf, which is rich in polyphenols and polysaccharides, has been rarely used. This study aimed to investigate the effect of *Eucommia ulmoides* leaf extracts (EULEs) on oxidative stress and meat quality of broilers. A total of 252 broilers were randomly divided into 3 treatments and fed with a control basal diet (CON), or a diet containing 250 mg/kg or 1,000 mg/kg of EULE for 51 days. Results showed that dietary supplementation of 250 mg/kg EULE increased significantly the average daily gain of broilers in the early stage (1–21 days), while 250 mg/kg or 1,000 mg/kg of EULE decreased the feed conversion ratio in the whole period (*P* < 0.05). Supplementation of 250 mg/kg EULE reduced the level of MDA in the liver (*P* < 0.05), while 1,000 mg/kg EULE decreased the serum level of MDA (*P* < 0.05), and the HDL level in serum was increased by 250 mg/kg or 1,000 mg/kg EULE (*P* < 0.05). Additionally, 250 mg/kg EULE decreased abdominal fat ratio and serum triglyceride (TC) level in broilers, while 250 or 1,000 mg/kg of EULE reduced drip loss in breast muscle (*P* < 0.05), and 1,000 mg/kg EULE reduced the cooking loss in thigh muscle (*P* < 0.05). In conclusion, dietary supplementation of 250 mg/kg of EULE could attenuate oxidative stress and improve the growth performance and meat quality in broilers.

## Introduction

Medicinal herb plants have been used to treat various human diseases in China for thousands of years, and are widely used in cosmetics, foods, and animal husbandry with their function and composition being defined in recent years (1, 2). Phytochemicals, such as polyphenols, terpenes, and flavonoids are considered bioactive compounds with multiple biological effects including antioxidant, anti-inflammatory, antimicrobial, and anti-tumor (3–5). With the prohibition of using antibiotics in feed (6), plant extracts have been expected to replace antibiotics and thus become a hot spot in animal nutrition research (7). Earlier, various herbs have been proven to attenuate oxidative stress and improve meat quality in poultry (2, 8, 9). *Eucommia ulmoides*, a plant belonging to the monotypic family *Eucommiaceae*, is one of the oldest tonics in Chinese traditional medicine and is widely distributed in East Asian countries, including China, Korea, and Japan. Although various bioactive components, such as lignans, iridoids, phenolics, steroids, terpenoids, and flavonoids have been identified in *Eucommia ulmoides* (10), phenolics and flavonoids, such as chlorogenic acid, protocatechuic acid, and quercetin are considered the main active components in leaves. Previous studies have found that *Eucommia ulmoides* flavones (EUF) could promote growth performance, improve intestinal health, and reduce colonization of coliform bacteria and diarrhea index in weanling piglets (11). *Eucommia ulmoides* leaf extract (EULE) has also been reported to improve the carcass traits of growing-finishing pigs and exert a lipid-lowering effect by activating the AMPK-ACC pathway and regulating mRNA expression levels related to lipid metabolism (12). In the diquat-induced oxidative stress piglet model, the dietary supplementation of EUF could increase the protein expression of nuclear Nrf2 and Keap1, and improve the antioxidant capacity of piglets (13). Although the antioxidant effect of EULE has been reported in pigs, the effect of EULE on broiler chickens, which can be easily affected by oxidative stress, has been rarely reported. The present study attempted to investigate the effects of EULE on oxidative stress, growth performance, and meat quality of broilers, in order to provide references for the usage of EULE as a natural feed additive in broilers.

## Materials and Methods

### Samples of EULE Preparation and Composition Analysis

*Eucommia ulmoides* leaves provided by Zhangjiajie Hengxing Biotechnology Co., LTD. were naturally dried and crushed before being extracted with 75°C deionized water for 12 h (repeat extraction two times). After filtration, the extraction solution was concentrated *in vacuo* and then dried by spray drying tower with the inlet air temperature fixed at 150°C and outlet air temperature of 80°C. Total phenolic content in the extract was measured by the Folin–Ciocalteu assay (14), total polysaccharides were measured by the phenol sulfuric acid method, and the phenolic components were analyzed by using an high-performance liquid chromatography-electrospray tandem mass spectrometry (LC-ESI-MS/MS) system. The concentrations of total phenolic, total polysaccharides, and top 10 phenolic components of the EULE are shown in Supplementary Table 1.

### Experimental Design and Diets

The protocol of the animal experiment was approved by the Institutional Animal Care and Use Committee (IACUC) of Hunan Agricultural University. A total of 252 Wen's Tianlu black 5 broilers (1-day-old) were randomly divided into 3 treatments with 6 replicates for each treatment (14 broilers per replicate). The light-dark cycle relies entirely on artificial lighting. In the first week of the experiment, broilers were under controlled light with 23 h/day and then reduced to 1.5 h/week until the fourth week, and the light time was stable for 18 h/day until the end of the experiment. At the beginning of the experiment, the temperature of the chicken house was 37°C, then dropped by 0.4°C every day until 25°C, and was stabilized until the end of the experiment. Broiler chickens in the treatments were fed with a basal diet (control group, CON), a diet with 250 mg/kg of *Eucommia ulmoides* leaf extracts (EULE250 group) or a diet supplemented with 1,000 mg/kg of EULE (EULE1000 group). The experimental period was 51 days, the early stage was 1–21 days, and the later stage was 21–51 days. Two different types of diets (starter diet for 0–21 days and finisher diet for 22–51 days) were considered to meet the nutrient levels followed by NRC (1994) specification (Supplementary Table 2). Broiler chickens were housed in a grid fence (length 120 cm, width 120 cm, and height 60 cm), and each grid fence was set as a repeat. All broilers had free access to feed and water.

### Growth Performance

The body weight (BW) of broilers was individually measured on the morning of 1, 21, and day 51 after fasting overnight, and feed intake per pen was collected daily throughout the trial to calculate average daily gain (ADG), average daily feed intake (ADFI), and feed conversion ratio (FCR).

### Sample Collections

In total, six broilers (one with average BW from each replicate pen) from each group were slaughtered by asphyxiation in a CO_2_ atmosphere. Blood samples (10 ml) were collected from the jugular vein into anticoagulant-free vacuum tubes and centrifuged at 1,500 × *g* for 10 min after standing at room temperature for 30 min to obtain the serum before storage at −80°C, until further analyses. After blood sampling, the feathers, cuticle of the foot, toe shell, and beak shell were removed and weighed, and then the organs and tissues were dissected and separated. The liver tissue was homogenized in physiological saline (1:9, m/v) at 4°C with a homogenizer (JXFSTPRP-24, Shanghai jingxin Experimental Technology, Shanghai, China), and liquid supernatant was collected after centrifuging at 3,000 *g* for 5 min.

### Measurement of Biochemical Indices in Serum and Liver

The total antioxidant capacity (T-AOC), total superoxide dismutase (T-SOD) activity, glutathione peroxidase (GSH-PX) activity, and malondialdehyde (MDA), an indicator of oxidative stress, were determined in serum and the liver by using respective assay kits (Nanjing Jiancheng Bioengineering Institute, Nanjing, China) according to the manufacturer's instructions as described previously (15). Triglyceride (TC), total cholesterol (TG), high-density lipoprotein (HDL), and low-density lipoprotein (LDL) were detected in serum by using respective assay kits (Nanjing Jiancheng Bioengineering Institute, Nanjing, China) according to the manufacturer's instructions as described previously by Wu et al. (15). All the above indices were determined by a porous chemiluminescence instrument (Varioskan flash, ThermoFisher Scientific, USA).

### Measurement of Organ Index and Slaughtering Performance

On the morning of day 51, one bird per replicate (*n* = 6) close to the average BW was slaughtered in the CO_2_ atmosphere, the viscera were collected (liver, thymus, spleen, and bursa), and then the organ index (organ weight/body weight ^*^ 100%) was calculated. After the chicken is executed, the slaughter weight was obtained by removing the feathers, the cuticles of the feet, the toe shell, and the beak shell. On this basis, by removing the weight of the trachea, esophagus, crop, intestine, spleen, pancreas, gallbladder, heart, liver, glandular stomach, muscle stomach, lung, abdominal fat, head, foot, and reproductive organs, the weight of the carcass is obtained. According to the “poultry production performance terms and measurement statistical methods” (NY/T 823-2004) (16), the slaughter rate, eviscerated rate, breast muscle rate, leg muscle rate, and abdominal fat rate were calculated. The index calculation formula is as follows:

breast muscle rate (%) = breast muscle weight/carcass weight ^*^ 100

Leg muscle rate (%) = leg muscle weight/carcass weight ^*^ 100

Abdominal fat rate (%) = abdominal fat weight/full net bore weight ^*^100

Slaughter rate (%) = slaughter weight/live weight ^*^ 100

Full bore rate (%) = carcass weight/live weight ^*^ 100

### Measurement of Meat Quality

The pH values at 45 min and 24 h postmortem of meat sample (breast muscles and leg muscles) were measured at three locations *via* a portable pH meter (205, Testo, Germany). After slaughtering, a 5-g meat sample of similar size was taken for water drop loss, then placed inside the refrigerator at 4°C for 24 h, and later taken out and weighed again. The drip loss was calculated using the following formula: Drip loss (%) = 100 × (initial weight - final weight)/initial weight. Similarly, a 20-g meat sample of similar size was taken for water cooking loss, then vacuum-sealed using a sealed bag, placed in a water bath pot, heated to a central temperature of 70°C, and then taken out after absorbing the water and weighed again. The cooking loss was calculated by the following formula: cooking loss (%) = 100 × (initial weight - final weight)/initial weight. The color evaluation procedure was based on the determination of Hunter values L^*^ (whiteness/darkness), a^*^ (redness/greenness), and b^*^ (yellowness/blueness).

### Statistical Analysis

The results were expressed as means ± standard deviation (SD). The significant differences between the groups were analyzed by one-way analysis of variance tests, followed by Fisher's least significant difference (LSD) and Duncan's multiple range tests with the SPSS statistical program (SPSS19, IBM Corp., Armonk, NY, USA). A probability of *p* < 0.05 was considered significant.

## Results

### The Effect of EULEs on the Growth Performance of Broilers

As shown in Table 1, the supplementation of 250 mg/kg EULEs significantly increased the ADG and final BW of broilers as compared with the control group from 1 to 21 days of age (*p* < 0.05). Moreover, the dietary supplementation of 250 mg/kg or 1,000 mg/kg EULEs significantly decreased the FCR (*p* < 0.05) in the whole experimental stage.

**Table 1:** Effects of *Eucommia ulmoides* leaf extracts (EULEs) on the growth performance of broilers.

**Items**	**Indices**	**Groups**			***P*** **-value**
		**CON**	**EULE250**	**EULE1000**	
Early stage (day 0–day 21)	Initial BW (g)	43.60 ± 0.90	42.40 ± 1.25	42.40 ± 1.25	0.100
	ADG (g)	11.80 ± 1.11^a^	13.70 ± 1.24^b^	12.10 ± 1.31^a^	0.039
	ADFI (g)	32.60 ± 2.96	31.57 ± 0.90	31.57 ± 0.73	0.577
	FCR (g/g)	2.77 ± 0.34	2.32 ± 0.19	2.65 ± 0.36	0.057
	Final BW (g)	291.62 ± 23.20^a^	329.47 ± 25.04^b^	296.96 ± 27.79^a^	0.043
Finishing stage (day 21–day 51)	ADG (g)	25.51 ± 1.29	26.81 ± 1.46	26.50 ± 1.39	0.267
	ADFI (g)	107.85 ± 6.08	105.14 ± 3.69	102.39 ± 9.85	0.424
	FCR (g/g)	4.23 ± 0.11^a^	3.93 ± 0.25^b^	3.86 ± 0.24^b^	0.019
	Final BW (g)	1056.99 ± 54.92	1133.77 ± 30.91	1091.82 ± 68.31	0.076
Whole period (day 0–day 51)	ADG (g)	19.87 ± 1.07	21.40 ± 0.59	20.56 ± 1.33	0.069
	ADFI (g)	76.84 ± 3.36	74.84 ± 2.32	73.23 ± 5.76	0.333
	FCR (g/g)	3.87 ± 0.12^a^	3.50 ± 0.14^b^	3.56 ± 0.20^b^	0.002

*Data represent the mean value of six samples per treatment. CON, a basal diet; EULE250, a basal diet supplemented with 250 mg/kg EULEs. EULE1000, a basal diet supplemented with 1,000 mg/kg EULEs. BW, body weight; ADG, average daily gain; ADFI, average daily feed intake; FCR, feed conversion ratio. Data are shown as means ± SD. In the same row, values with different small letter superscripts mean a significant difference (p < 0.05)*.

### The Effect of EULEs on Antioxidant Indicators in the Serum and the Liver

To understand the effect of EULE on the antioxidant status of broilers, antioxidant indicators, such as T-SOD, T-AOC, GSH-Px, and MDA were then measured. As shown in Table 2, the serum level of MDA, an indicator of lipid peroxidation, was significantly decreased in the EULE250 group (*p* < 0.05), as compared with the CON group. However, there were no significant differences in the serum levels of T-AOC, T-SOD, and GSH-Px among the three groups. Consistently, the level of MDA in the liver was decreased in EUEL250 (*p* < 0.05 compared with the CON group), while no significant differences were observed in T-AOC, T-SOD, and GSH-Px levels in the liver of broilers among the three groups.

**Table 2:** Effects of EULEs on antioxidant parameters in the serum and the liver.

**Item**	**Groups**			***P*** **-value**
	**CON**	**EULE250**	**EULE1000**	
serum				
T-AOC (U/ml)	6.70 ± 1.55	5.55 ± 1.27	7.27 ± 1.53	0.151
SOD (U/ml)	53.12 ± 1.29	51.21 ± 5.03	51.31 ± 1.22	0.499
GSH-PX (U/mL)	1,738.36 ± 329.30	1,829.40 ± 521.01	1,926.66 ± 315.64	0.721
MDA (nmol/ml)	4.14 ± 0.78^a^	3.96 ± 0.84^a^	3.06 ± 0.25^b^	0.033
liver				
T-AOC (U/mg protein)	0.12 ± 0.02	0.12 ± 0.01	0.10 ± 0.03	0.276
SOD (U/mg protein)	0.51 ± 0.04	0.56 ± 0.11	0.57 ± 0.17	0.730
GSH-PX (U/mg protein)	156.51 ± 18.39	162.12 ± 18.99	152.65 ± 57.95	0.905
MDA (U/mg protein)	0.097 ± 0.018^a^	0.073 ± 0.017^b^	0.097 ± 0.021^a^	0.044

*Data represent the mean value of six samples per treatment. CON, a basal diet; EULE250, a basal diet supplemented with 250 mg/kg EULEs. EULE1000, a basal diet supplemented with 1,000 mg/kg EULEs. T-AOC, total antioxidant capacity; SOD, superoxide dismutase; GSH-Px, glutathione peroxidase; MDA, malondialdehyde. Data are shown as means ± SD. In the same row, values with different small letter superscripts mean a significant difference (p < 0.05)*.

### The Effect of EULEs on Serum Lipids in Broilers

Indices, such as TC, TG, HDL, and LDL were next measured to reflect serum lipids in broilers. As shown in Table 3, the serum level of TG in the EULE250 group was significantly decreased (*p* < 0.001) as compared with the control group, while the HDL level in both the EULE250 and EULE1000 groups was significantly increased (*p* < 0.05). However, there were no significant differences in the levels of TC and LDL among the three groups.

**Table 3:** Effect of the EULEs on serum lipids in broilers.

**Item**	**Group**			***P*** **-value**
	**CON**	**EULE250**	**EULE1000**	
TG(mmol/L)	0.23 ± 0.062^a^	0.11 ± 0.034^b^	0.28 ± 0.072^a^	0.001
TC(mmol/L)	3.14 ± 0.25	3.27 ± 0.38	3.17 ± 0.25	0.725
HDL(mmol/L)	7.38 ± 1.17^a^	9.02 ± 0.84^b^	8.64 ± 0.68^b^	0.020
LDL(mmol/L)	0.62 ± 0.59	0.55 ± 0.073	0.59 ± 0.12	0.379

*Data represent the mean value of six samples per treatment. CON, a basal diet; EULE250, a basal diet supplemented with 250 mg/kg EULEs. EULE1000, a basal diet supplemented with 1,000 mg/kg EULEs. TG, triglyceride; TC, total cholesterol; HDL, high-density lipoprotein cholesterol; LDL, low-density lipoprotein cholesterol. Data are shown as means ± SD. In the same row, values with different small letter superscripts mean a significant difference (p < 0.05)*.

### The Effects of the EULEs on the Slaughter Performance of Broilers

Indices of the slaughter performance are shown in Table 4, the dietary supplementation of 250 mg/Kg EULE significantly decreased the abdominal fat yield (*p* < 0.05) as compared with the CON group. However, no significant differences were observed in dressing percentage, eviscerated carcass yield, breast yield, and thigh yield among the three groups.

**Table 4:** Effects of EULEs on the slaughter performance of broilers.

**Item (%)**	**Group**			***P*** **-value**
	**CON**	**EULE250**	**EULE1000**	
Dressing percentage	91.85 ± 2.29	91.00 ± 1.26	92.22 ± 1.98	0.533
Eviscerated carcass yield	71.34 ± 8.37	75.54 ± 3.69	76.30 ± 1.53	0.257
Breast yield	14.15 ± 4.75	12.91 ± 0.98	12.38 ± 1.73	0.581
Thigh yield	19.22 ± 2.61	17.14 ± 1.35	16.90 ± 0.82	0.072
Abdominal fat yield	1.11 ± 0.38^a^	0.70 ± 0.18^b^	1.46 ± 0.32^a^	0.002

*Data represent the mean value of six samples per treatment. CON, a basal diet; EULE250, a basal diet supplemented with 250 mg/kg EULEs. EULE1000, a basal diet supplemented with 1,000 mg/kg EULEs. Data are shown as means ± SD. In the same row, values with different small letter superscripts mean a significant difference (p < 0.05)*.

### The Effect of EULEs on the Meat Quality of Broilers

As shown in Table 5, the dietary supplementation of 250 or 1,000 mg/kg EULEs significantly decreased the drip loss in the breast muscle as compared with the CON group (*p* < 0.05). Moreover, the dietary supplementation of 1,000 mg/kg EULEs significantly decreased the cooking loss of the thigh muscle (*p* < 0.05) in broilers as compared with the CON group, but there were no significant differences in parameters of the meat quality including drip loss and meat color among the three groups.

**Table 5:** Effect of EULEs on the meat quality of broilers.

**Item**	**Groups**			***P*** **-value**
	**CON**	**EULE250**	**EULE1000**	
Breast muscle				
pH at 45 min	6.00 ± 0.31	5.93 ± 0.25	6.03 ± 0.29	0.832
pH at 24 h	5.83 ± 0.12	5.76 ± 0.13	5.89 ± 0.10	0.197
L^*^	45.6 ± 3.32	45.35 ± 2.63	46.79 ± 3.21	0.691
a^*^	3.86 ± 1.28	4.12 ± 1.09	5.69 ± 2.08	0.118
b^*^	5.84 ± 1.72	4.96 ± 1.00	6.65 ± 1.90	0.215
Drip loss (%)	2.57 ± 0.56^a^	1.75 ± 0.57^b^	0.97 ± 0.32^c^	<0.001
Cooking loss (%)	19.43 ± 3.35	16.02 ± 2.57	18.76 ± 3.45	0.173
Thigh muscle				
pH at 45min	5.96 ± 0.11	6.28 ± 0.34	6.13 ± 0.50	0.317
pH at 24h	6.27 ± 0.12	6.22 ± 0.22	6.25 ± 0.17	0.884
L^*^	46.32 ± 4.17	49.43 ± 4.31	44.24 ± 3.19	0.103
a^*^	9.09 ± 1.82	9.69 ± 2.11	10.97 ± 1.87	0.263
b^*^	7.13 ± 1.29	7.17 ± 0.59	6.79 ± 0.55	0.713
Drip loss(%)	1.23 ± 0.18	0.82 ± 0.79	1.31 ± 0.39	0.239
Cooking loss(%)	22.28 ± 5.08^a^	21.87 ± 3.13^a^	14.5 ± 4.94^b^	0.014

*Data represent the mean value of six samples per treatment*.

L^*^, lightness; a^*^, redness; b^*^, yellowness.

*Data were shown as means ± SD. In the same row, values with different small letter superscripts mean a significant difference (P < 0.05)*.

### The Effect of EULEs on Organ Indexes in Broilers

As shown in Table 6, the dietary supplementation of 250 or 1,000 mg/kg EULEs had no significant influence on organ indices.

**Table 6:** Effects of EULEs on organ indices in broilers.

**Item** **(% body weight)**	**Group**			***P*** **-value**
	**CON**	**EULE250**	**EULE1000**	
Liver	2.12 ± 0.14	1.95 ± 0.15	2.03 ± 0.17	0.169
Spleen	0.19 ± 0.06	0.16 ± 0.03	0.15 ± 0.02	0.246
Thymus	0.23 ± 0.1	0.29 ± 0.08	0.29 ± 0.1	0.476
Bursa	0.34 ± 0.06	0.31 ± 0.09	0.31 ± 0.06	0.711

*Data represent the mean value of six samples per treatment. CON, a basal diet; EULE250, a basal diet supplemented with 250 mg/kg EULEs. EULE1000, a basal diet supplemented with 1,000 mg/kg EULEs. Data are shown as means ± SD*.

## Discussion

The dietary supplementation of plant extracts, such as chestnut wood extract, achyranthes japonica extract, and Illicium verum extracts, has been extensively used for improving growth performance, immunity, and antioxidant activity to prevent infectious diseases in broiler chickens and pigs (17–19). *Eucommia ulmoides* leaf is a medicinal herb widely used in traditional Chinese medicine and possesses potential pharmacological effects involving antioxidant, anti-inflammation, and lipid-modulating (20). It has been reported that the dietary supplementation of chlorogenic acid-enriched extract from *Eucommia ulmoides* leaf could increase ADG and reduce the FCR of broilers under heat stress (21), and increase the ADG of weaned piglets (22). Sato et al. (23) revealed that the dietary supplementation of polyphenolic extract from *Eucommia ulmoides* Oliver leaf increased final BW and ADG, but reduced FCR in finishing pigs. *Eucommia ulmoides* has also been reported to inhibit pro-inflammatory responses by modulating mitogen-activated protein kinases (MAPKs), phosphatidylinositol 3-kinase/protein kinase B (PI3K/Akt), and glycogen synthase kinase 3-beta (GSK-3β) with suppressed nuclear factor kappa B (NF-κB) activation and Nrf2-dependent HO-1 activation (24). In the present study, the dietary supplementation of 250 mg/kg EULEs significantly increased ADG and BW of broilers in the early period (day 1–21), while 250 or 1,000 mg/kg EULE decreased FCR in the whole period (day 1 or 51). These results indicated that EULE has beneficial effects on the growth performance of broilers.

As fat is the primary energy storage material in the body, fat synthesis and oxidation are directly related to the level of energy metabolism. Poultry has a high energy metabolism rate, and its abdominal fat deposition rate is higher than the rate of weight gain (25). However, the abdominal fat rate of poultry is kept high in the long-term commercial breeding, which affects the carcass quality of poultry and reduces the feed conversion rate (26). In this study, the supplementation of 250 mg/kg EULEs significantly reduced abdominal fat deposition with decreased serum TG level, which is considered the key factor that affects fat deposition in animals (27). The results of HPLC analysis in this study showed that chlorogenic acid, which accounted for 21.46% of the total polyphenols, was the main polyphenol in EULE. Previous studies also indicated that *Eucommia ulmoides* leaves contain polyphenols and flavonoids, such as chlorogenic acid, quercetin, and protocatechuic acid (28), and these phenolic components exert beneficial effects on lipid metabolism by attenuating adipogenic differentiation through their interaction with peroxisome proliferators-activated receptor-α2 (PPARγ) and AMP-activated protein kinase (AMPK) (29, 30). Thus, polyphenols of EULE at least partly played a role in regulating lipid metabolism and TC production in broilers.

External stimulation, feed pollution, or deterioration can increase reactive oxygen species (ROS) production to induce oxidative stress and have adverse effects on the broilers' growth and development. Low concentrations of ROS are essential for the normal physiological activity of cells, but excessive ROS production may destroy lipids and cause damage to cells (31, 32). The content of MDA is usually used to evaluate the degree of lipid oxidation (33). Chlorogenic acid-enriched extract from *Eucommia ulmoides* leaf has been reported to increase the activity of antioxidant enzymes and decrease the MDA level in broilers (21) and ducks (34). The main phenolic components of EULE, such as chlorogenic acid, protocatechuic acid, and quercetin, have also been reported to protect the body against oxidative damage (35–37). In addition, Gao et al. (38) have demonstrated that *Eucommia ulmoides* polysaccharide (EUP) can reduce oxidative stress damage by reducing excessive ROS and MDA production caused by hepatic ischemia-reperfusion (I/R). In this study, the supplementation of EULE reduced the MDA level in both serum and liver of broilers, which indicates that EULE has an alleviating effect on oxidative stress. Other studies have reported that the water extract of *Eucommia ulmoides* may act as a ROS scavenger (39).

Moreover, the supplementation of 250 or 1,000 mg/kg EULE reduced the drip loss of breast meat, and the supplementation of 1,000 mg/kg EULE decreased the cooking loss of thigh muscle in broilers in the present study, suggesting that EULE may improve the water-holding capacity of meat. Similarly, Liu et al. also found that the dietary supplementation of extract from *Eucommia ulmoides* leaves could improve the cooking loss of mutton caused by transportation stress (40). Lipid oxidation in meat starts immediately after slaughtering and continues during post-mortem aging (41), it changes membrane structure and functions of the muscle cell and accelerates water-holding capacity loss (42). Meanwhile, secondary products of lipid oxidation can react with proteins, peptides, and amino acids to accelerate protein oxidation, which result in changes in the protein or peptide structure, leading to the loss of functionality or biological function and reducing water-holding capacity (43, 44). As a candidate antioxidant for meat and meat products, polyphenols can be used as ROS scavengers, lipoxygenase inhibitors, and reducing agents for metmyoglobin to inhibit the oxidation process (45–47). Thus, the antioxidant capacity of polyphenols in EULE may at least partly contribute to the water-holding capacity of meat.

## Conclusion

In conclusion, dietary supplementation with 250 mg/kg EULE can increase the average daily gain of broilers in the early stage, and 250 or 1,000 mg/kg EULEs can decrease the feed conversion ratio over the entire period. Additionally, 250 mg/kg EULEs can reduce the abdominal fat ratio and serum triglyceride level in broilers, with a decreased MDA level in the liver and drip loss in breast muscle, while 1,000 mg/kg EULEs only reduce the drip loss in breast muscle and cooking loss in the thigh muscle. Our results confirmed that 250 mg/kg EULEs improved the growth performance, meat quality, and antioxidant response of broilers.

## Data Availability Statement

The raw data supporting the conclusions of this article will be made available by the authors, without undue reservation.

## Author Contributions

JY, RH, JT, and XW designed the experiments. JY and BL conducted the experiments. YW and ZT helped with animal experiments. JY analyzed the data and wrote the original draft. CF and XW revised the manuscript. JH reviewed and edited. All authors read and approved the final manuscript. All authors contributed to the article and approved the submitted version.

## Funding

This work was supported by the National Natural Science Foundation of China (31972600 and 31772819).

## Conflict of Interest

The authors declare that the research was conducted in the absence of any commercial or financial relationships that could be construed as a potential conflict of interest.

## Publisher's Note

All claims expressed in this article are solely those of the authors and do not necessarily represent those of their affiliated organizations, or those of the publisher, the editors and the reviewers. Any product that may be evaluated in this article, or claim that may be made by its manufacturer, is not guaranteed or endorsed by the publisher.
