# Supplementary material for: Effect of Eucommia ulmoides Leaf Extract on Growth Performance, Carcass Traits, Parameters of Oxidative Stress, and Lipid Metabolism in Broiler Chickens
Source: Front Vet Sci. 2022 Jul 28;9:945981. doi: 10.3389/fvets.2022.945981 (PMC9371477; doi:10.3389/fvets.2022.945981)
Supplement: Supplementary file 1 [file Table_1.docx]

**Supplemental Table 1.** Phenolic components of *Eucommia ulmoides* leaf extracts

| Items | percent％ |
| --- | --- |
| Total Phenolics | 8.67 |
| Total Polysaccharides | 2.49 |
| Phenolic components ranking* |  |
| Chlorogenic acid | 21.46 |
| Amurensin | 4.05 |
| Isochlorogenic acid | 3.80 |
| o-Coumaric acid-beta-D-glucoside | 1.12 |
| (-)-Olivil-4''-O-beta-D-glucopyranoside | 0.39 |
| Quercetin-3-L-arabino-7-D-glucoside | 0.36 |
| 3-Carboxy-4-hydroxy-phenoxy glucoside | 0.21 |
| Sodium ferulate | 0.20 |
| Methyl di-alpha-L-rhamnoside | 0.17 |
| Triptofordin A | 0.15 |

*Proportion of the top 10 phenolic components.
