# Supplementary material for: Effect of Eucommia ulmoides Leaf Extract on Growth Performance, Carcass Traits, Parameters of Oxidative Stress, and Lipid Metabolism in Broiler Chickens
Source: Front Vet Sci. 2022 Jul 28;9:945981. doi: 10.3389/fvets.2022.945981 (PMC9371477; doi:10.3389/fvets.2022.945981)
Supplement: Supplementary file 2 [file Table_2.docx]

**Supplemental Table 2.** Nutrient composition of basal diets^1^

| Items | Phase 1 | Phase 2 |
| --- | --- | --- |
|  | 1-21d | 21-51d |
| Ingredients |  |  |
| Corn | 56.48 | 57.85 |
| Soybean meal | 29.63 | 28.22 |
| Corn gluten meal | 0.70 | 2.5 |
| Wheat bran | 4.60 | 2.5 |
| Soy oil | 4.40 | 5 |
| L-Lysine | 0.26 | 0.22 |
| Methionine | 0.29 | 0.26 |
| Threonine | 0.09 | 0.05 |
| CaHPO_4_ | 1.70 | 1.75 |
| Limestone | 1.36 | 1.15 |
| Vitamin-mineral premix ^2^ | 0.48 | 0.5 |
| Total | 100.00 | 100.00 |
| Nutrient levels ^3^ |  |  |
| Metabolizable energy /( MJ /kg) | 12.54 | 12.97 |
| Crude protein | 19.77 | 19.58 |
| Calcium | 1.00 | 0.92 |
| Total P | 0.66 | 0.65 |
| Lysine | 1.16 | 1.10 |
| Methionine | 0.59 | 0.57 |
| Tryptophan | 0.19 | 0.18 |
| Threonine | 0.80 | 0.77 |

^1^ 250mg or 1000mg *Eucommia ulmoides* leaf extract were fully mixed with 1kg basal diet.

^2^ The premix provided the following per kilogram of diet: zinc, 83 mg; iron, 90 mg; manganese, 83 mg; copper, 10 mg; iodine, 0.34 mg; selenium, 0.3 mg; vitamin A, 10,000 IU; vitamin D, 3000 IU; vitamin E, 30 IU; vitamin K3, 2.3 mg; vitamin B12, 1.35 mg; riboflavin, 6.3 mg; nicotinic acid, 30 mg; pantothenic acid, 13 mg; pyridoxine, 3.4 mg; biotin, 0.22 mg; and choline chloride, 850 mg.

^3^ These nutrient levels were calculated from data provided by NRC (1994).
